# Comparative recruitment, morphology and reproduction of a generalist trematode, *Dicrocoelium dendriticum*, in three species of host

**DOI:** 10.1017/S0031182015000621

**Published:** 2015-06-10

**Authors:** MELISSA A. BECK, CAMERON P. GOATER, DOUGLAS D. COLWELL

**Affiliations:** 1 Department of Biological Sciences, University of Lethbridge, 4401 University Drive, Lethbridge, Alberta, CanadaT1K 3M4; 2 Agriculture and Agri-Food Canada, Lethbridge Research Station, 5403 1stAve South, Lethbridge, Alberta, CanadaT1J 4B1

**Keywords:** Parasite reproduction, comparative fluke performance, fitness

## Abstract

Epidemiological rate parameters of host generalist parasites are difficult to estimate, especially in cases where variation in parasite performance can be attributed to host species. Such cases are likely common for generalist parasites of sympatric grazing mammals. In this study, we combined data from experimental exposures in cattle and sheep and natural infections in elk to compare the recruitment, morphology and reproduction of adult *Dicrocoelium dendriticum*, a generalist trematode that has emerged in sympatric grazing hosts in Cypress Hills Provincial Park, Alberta. Overall, there were no significant differences in the recruitment of metacercariae and in the pre-patency period of adults in experimentally exposed cattle and sheep. All flukes reached reproductive maturity and the degree of reproductive inequality between individual flukes within each infrapopulation was moderate and approximately equal among the three host species. Neither fluke size nor *per capita* fecundity was constrained by density dependence. Thus, fitness parameters associated with growth and reproduction were approximately equivalent among at least three species of definitive host, two of which are sympatric on pastures in this Park. The generalist life-history strategy of this trematode, which is known to extend to other stages of its life cycle, has likely contributed to its invasion history outside its native range in Europe.

## INTRODUCTION

Parasitologists have long recognized that a parasite's position along the host specialist – host generalist continuum influences fundamental aspects of host/parasite biology. Thus, the extent to which a parasite is a host specialist or host generalist impacts the nature of host immunological responses, the magnitude of transmission asymmetries among host species, and also influences key components of parasite fitness, such as growth, reproduction and development (Combes, 2001; Hudson *et al*. 2002; Poulin, 2007). The direction and magnitude of parasite-mediated natural selection and parasite–host co-evolution can also be expected to differ for host specialists *vs* generalists (e.g. Richner, 1998). Perhaps less well recognized is the notion that the degree of host specialization also has important implications for applied and theoretical epidemiologists. For parasites that are host specialists, key rate parameters such as transmission, growth, reproduction, senescence and parasite-induced host mortality can be estimated from experiments involving a single (or few) species of host. It follows that control strategies will be much simpler in cases involving single species of parasite in a single species of host. But the epidemiological picture becomes much more complex for generalist parasites that have the potential to infect multiple species of sympatric host (Grenfell *et al.* 2002). In an early example, Hairston (1962) showed that the trematode *Schistosoma japonicum* developed within sympatric humans, dogs, pigs and rats. Within each of these hosts, parameters such as prevalence, fluke intensity, fluke size and *per capita* fluke reproduction varied extensively. He then used simple models to demonstrate that effective control was only realistic if intervention was targeted at the rat host population. This example illustrates that for generalist parasites in multiple species of host, the relative performance of individual parasites in different hosts is a key epidemiological factor.

The relative performance of generalist parasites within multi-host communities is particularly important in the context of parasites that have been introduced outside their native host ranges. The rate of parasite ‘spillover’ between hosts that have invaded a new habitat and sympatric species of host may be high (Cleaveland *et al.* 2002; Cross *et al*. 2009), but it is only epidemiologically important if the invasive parasites develop to maturity within their new hosts. Further, the extent to which the presence of one or more species of host within a multi-host community either dilutes or enhances (Keesing *et al.* 2006) overall rates of transmission into the host community will depend on parasite establishment and performance in different species of host. This means that the epidemiological consequences of invasion into a multi-host community will be determined by the rate of transmission of infective stages into different species of hosts, and also by variability in relative rates of development of individual flukes among sympatric host species. The problem is that for generalist parasites in multi-host systems, including those that involve invasive parasites, information on relative fluke performance in different sympatric species of host is rarely available. For the few exceptions involving invasive parasites or hosts (e.g. the trematode *S. japonicum* in four species of mammal (Hairston, 1962); the acanthocephalan *Metechinorhynchus salmonis* in ten species of fish (Holmes *et al.* 1977)), transmission dynamics within multi-host communities was strongly influenced by the relative performance of parasites within different species of host.

The liver fluke, *Dicrocoelium dendriticum* (Trematoda: Dicrocoeliidae) has been widely introduced outside its homeland in Central Europe, most likely through the movement of infected livestock (especially sheep) across international borders. The fluke is now common in Northern and Southern Europe, in Northern Africa, and in isolated pockets in Western and Eastern North America (Otranto and Traversa, 2002; Rojo-Vásquez *et al.* 2012). Evidence from the host survey literature indicates that the adult stage of *D. dendriticum* is a host generalist within and outside its native range, found in a wide array of cervine, ovine and bovine hosts. Furthermore, evidence from experimental infections in sheep, beef cattle and laboratory hamsters, together with fecal analyses for the characteristic eggs, has shown that at least some flukes reach reproductive maturity in all of these hosts (e.g. Manga-González *et al.* 1991; Campo *et al.* 2000; Sánchez-Campos *et al.* 2000; Manga-González and González-Lanza, 2005). Once absent in Alberta, Canada, the fluke is now common within a range of grazing mammals that co-graze pasture within Cypress Hills Interprovincial Park (CHP), a conservation reserve located in the southeastern corner of the province. Goater and Colwell (2007) showed that 60–90% of resident elk (*Cervus canadensis*) and beef cattle (*Bos taurus*) are infected from year to year and all infected hosts contain gravid flukes.

The broad host spectrum of this fluke and its recent introduction into CHP provides an opportunity to evaluate relative performance of a generalist parasite within a multi-host system. In this study, we used experimental exposures in sheep and cattle to compare relative patterns of fluke recovery and intensity and to obtain comparative data on *per capita* fluke size and reproduction. Opportunistic collections of hunter-shot elk provided comparable data for infected wildlife collected from CHP. Our overall aim is to evaluate the performance of flukes collected from alternative hosts to provide a foundation for subsequent assessment of the roles of alternative host species in the overall transmission of *D. dendriticum* eggs onto shared pasture.

## MATERIALS AND METHODS

### Cypress Hills Interprovincial Park

CHP is a 531,000 ha plateau rising approximately 200 m above the surrounding prairie (1050–1470 m above sea level) in Southeastern Alberta and Southwestern Saskatchewan, Canada. The southern perimeter of the park is located approximately 100 km north of the Canada/US border (49°37·5′N, 110°′W). Sympatric elk (*C. canadensis*), mule deer (*Odocoileus hemionus*), white-tailed deer (*Odocoileus virginianus*) and beef cattle graze within the park. A managed elk hunt, initiated in 1978, runs annually each fall to maintain a density of 350–700 animals (Hegel *et al.* 2009). Further details regarding the natural and cultural history of CHP are in Hildebrant and Hubner (1994).

### Experimental infections

A total of 18 (2 control and 16 experimental) weaned Canadian Arcott sheep (ages: 6 months to 9 years old) were selected from a research flock maintained at the Agriculture and Agri-Food Canada Lethbridge Research Centre (LRC) in Lethbridge, Alberta. Twelve Holstein cattle (ages: 6 months to 2 years old) were purchased from a dealer and housed at the LRC for the duration of the infection trials (two control and ten experimental). The steers and sheep were housed separately in a feedlot and fed a basic diet of hay/barley silage. All animals were handled and maintained under the guidelines specified by the Canada Council for Animal Care (LRC Animal Care Committee protocol numbers 08233, 0925 and 1044).

Prior to exposure to metacercariae, sheep and cattle were treated once with a standard application of Ivomec^®^ (Merial, Baie-D'Urfe, Quebec) to eliminate pre-existing helminth infections. For each infection trial, metacercariae were dissected from naturally infected formicid ants (*Formica sanguinea, F. subaenescens* and *F. fusca* (van Paridon and Goater, unpublished observations)) obtained from CHP in June 2011–2013 and packaged into gel capsules just prior to administration *per os*. Two sheep and two calves were assigned to Group A, the sham control, receiving capsules but no metacercariae. Group B (four sheep and four calves) were orally inoculated with 625 metacercariae in 2011. Twelve sheep and six calves (Group C) were inoculated with ~1000 metacercariae, half in 2012 and the others in a third trial in 2013 (Table 1).

**Table 1.** Summary of the number of adult *Dicrocoelium dendriticum* recovered from the livers of experimentally infected cattle and sheep with their associated fecal egg counts and number of eggs shed by flukes over for a 24 h period

Group	Infection dose	Host	*N*	Fluke intensity	Eggs/g feces	Mean number of egg/24 h
A	Control	Sheep	2	0	0	0
		Cattle	2	0	0	0
B	625	Sheep	4	137·3 ± 18·0	46·8 ± 7·8	1093·4 ± 204·7
		Cattle	4	42·8 ± 30·2	3·8 ± 2·8	1043·2 ± 206·5
C	1127 ± 186·1	Sheep	12	183·3 ± 27·7	35·8 ± 8·1	2256·0 ± 261·9
		Cattle	6	198·7 ± 47·5	11·2 ± 4·9	1986·6 ± 241·8

All values are mean ± s.e.m.

Rectal fecal samples were collected approximately every 2 weeks for up to 22 weeks post-infection (p.i.) to determine the approximate onset of fluke reproduction in individual hosts. Fecal samples were labelled and frozen prior to analyses. Fecal egg counts were conducted using the modified Wisconsin method as outlined by Zajac and Conboy (2012) with saturated zinc sulphate solution (specific gravity = 1·36).

Animals were selected at random for slaughter at 110–150 days p.i. Live flukes were collected from sheep and cattle using the standard methods described in Goater and Colwell (2007). Each liver was weighed and then cut into approximately 5 mm-wide strips. Each strip was mechanically palpated into saline to dislodge the flukes from the bile duct. Live adult flukes were counted under a dissecting microscope and collected for subsequent analyses. These procedures allowed us to compare patterns of adult fluke recovery, mean fluke intensity and fluke fecundity (Bush *et al*. 1997) in the two species of host.

### Natural infections in elk

Logistical constraints prevented experimental exposures in wildlife. However, we collected data on fluke intensity and individual fluke fecundity (see below) in elk during the 2009–2013 hunting seasons to provide a general comparison with data on adult flukes collected at approximately the same time from sheep and cattle. Wherever possible, live adult flukes were collected for subsequent analyses in the laboratory.

### Morphology and reproduction of adult flukes

Data on *per capita* fluke size and reproduction came from live adult flukes collected at necropsy from five randomly selected sheep and cattle, and three elk harvested in 2013. Flukes from sheep and cattle were between 110 and 150 days old. The ages of flukes originating from elk are unknown, but were assumed to be equal to, or greater than, their 60-day pre-patency period (review by Otranto and Traversa, 2002). All intact flukes from each host were removed and washed with RPMI 1640 culture media (Sigma-Aldrich Canada Co., Oakville, Ontario) at pH 7·4. A subset of adult flukes was randomly selected from each host, isolated and then transferred immediately into 24-well tissue culture plates. Each well was filled with 3 mL of RPMI 1640 and the plates were incubated at 37 °C for 24 h to simulate host body temperature. This procedure is standard for the maintenance of live parasites for prolonged periods (Geary *et al.* 1993). Adult *D. dendriticum* continually shed eggs for up to 4 days within this medium (Beck and Goater, unpublished observations). Following a 24 h incubation period, the flukes were fixed in heated aceto-formal-alcohol (AFA) under light coverslip pressure.

These incubation and preservation procedures provided flukes of excellent quality. Morphological data on body length (BL), body surface area (BA) and uterus area (UA) were obtained from digital images (resolution of 250 pixels mm^−1^) using ImageJ software (Abramoff *et al.* 2004) for flukes from sheep (*N* = 5–18 flukes/host, total *N* = 80), cattle (*N* = 7–11 flukes/host, total *N* = 47) and elk (*N* = 6–25 flukes/host, total *N* = 39) following procedures adapted from Valero *et al*. (1999) for liver flukes (*Fasciola hepatica*) originating from naturally infected sheep. The fecundity of individual flukes was determined by counting the numbers of eggs at the bottom of each well for sheep (*N* = 85), cattle (*N* = 51) and elk (*N* = 54). Following these counts, flukes and eggs were preserved in 90% ethanol. To estimate total reproductive output/day, *ex vivo* egg counts over 24 h were averaged for each host species and multiplied by fluke intensity to estimate total eggs shed per 24 h.

### Statistical analyses

Parasitological terms such as intensity and infrapopulation follow definitions in Bush *et al*. (1997). Parametric tests involving intensity and egg count data were used with assumptions of normality met using raw or square root (*n* + 1) transformed data. Differences in mean values were evaluated using independent *t*-tests or ANOVAs with Tukey's *post hoc* analyses. Pairwise comparisons of proportions used chi-square (*χ*^2^) tests. 95% confidence intervals (CI) were calculated for proportions (*p*) using the Wald method [Vollset, 1993; *p* ± *z*√(*pq*/*n*), where *z* = 1 − *α*/2 of the standard normal distribution and *q* = 1 − *p*].

Differences in rates of maturation and fluke recovery in experimentally infected cattle and sheep were evaluated using data on mean time to egg shedding and proportion of recovered flukes. Proportion of recovered flukes refers to the numbers of recovered adult flukes relative to the numbers of metacercariae administered. Assessment of density dependence in fluke growth and reproduction within each host species was evaluated with standard parametric regressions involving fluke intensity, fluke BA, and *ex vivo* egg counts. Morphometric data were evaluated with a nested ANOVA to apportion variation in fluke BA into between-species, between individual hosts and between individual fluke effects. The percentage contribution by host species and individual hosts were calculated as a percentage of total variance (Rowe *et al.* 2008).

Comparisons of fluke reproduction included parametric comparisons of EPG on the date of necropsy and *ex vivo* egg counts from individual live flukes obtained during necropsy. A nested ANOVA design was used to apportion observed variation into between-species, between individual hosts and between individual fluke effects. Percentage contribution of each main effect was calculated as a percentage of total variance. Lorenz curves and Gini coefficients, adjusted for sample sizes, were then used to describe inequality in *ex vivo* fluke reproduction within infrapopulations of the three different definitive hosts according to methods described in Dobson (1986). Applied examples included Shostak and Dick (1987) and Poulin and Latham (2002). Prior to the estimation of reproductive inequalities, *ex vivo* egg counts were ranked from lowest to highest for each host species. Lorenz curves are then calculated by plotting cumulative per cent (%) of daily reproductive output against the cumulative number of individual flukes.

## RESULTS

### Rates of maturation and fluke recovery in sheep and cattle

Fluke eggs were first detected in lamb feces at 7–9 weeks p.i., whereas eggs were first detected in cattle feces at 8–12 weeks p.i. Although these data indicate earlier egg release in sheep, there was no significant difference in the timing of initial egg release between the two species of host (*t*_20_ = −1·11, *P* = 0·28). Flukes were present within the livers of all exposed hosts at necropsy (Table 1). Intensities ranged from 46 to 283 flukes in sheep and 1 to 324 in cattle. The proportion of recovered adult flukes (sheep: 18·4%; 95% CI = 0·0–42·4%; and cattle: 12·8%; 95% CI = 0·0–33·5%) did not significantly differ between hosts (*χ*^2^ = 0·95, d.f. = 1, *P* = 0·33).

### Density-dependent fluke performance

The association between size of individual flukes, measured as BA, and *per capita* fluke fecundity was non-significant in sheep (*r* = 0·03, *F*_1,75_ = 0·05, *P* = 0·82) and elk (*r* = 0·05, *F*_1,37_ = 0·08, *P* = 0·78). This association was significantly positive for flukes from cattle (*r* = 0·34, *F*_1,45_ = 5·88, *P* = 0·02), indicating that larger flukes tended to produced more eggs. BA varied significantly with intensity in cattle (*r* = 0·38, *F*_1,45_ = 7·35, *P* < 0·01) but not in sheep (*r* = 0·05, *F*_1,78_ = 0·18, *P* = 0·67). *Ex vivo* egg counts did not vary significantly with fluke intensity in cattle (*r* = 0·08, *F*_1,49_ = 0·35, *P* = 0·56) or sheep (*r* = 0·18, *F*_1,81_ = 2·77, *P* = 0·10). Analyses comparing *ex vivo* egg counts could not be completed for elk because fluke intensity data were only available for two hosts.

### Comparative morphology of adult flukes in cattle, sheep and elk

Morphological data on fluke BA, UA and BL were highly positively and significantly inter-correlated for flukes assessed from each host species, particularly for data involving BA and UA (Table 2; Range in *R*^2^ values: BA *vs* UA = 0·48–0·88; BA *vs* UL = 0·62–0·88; BL *vs* UA = 0·41–0·58). Owing to the magnitude of these significant inter-correlations, and for consistency with the literature involving other flukes, we focused our comparative analyses on BA.

**Table 2.** Morphometric data for adult *Dicrocoelium dendriticum* from experimentally infected sheep and cattle and naturally infected elk from Cypress Hills Interprovincial Park, Alberta

	Sheep (*N* = 80)	Cattle (*N* = 47)	Elk (*N* = 39)	ANOVA *P*-value
Body area (BA; mm^2^)	6·6 ± 0·1	7·8 ± 0·3	6·7 ± 0·3	<0·001
	3·9–10·6	4·5–11·4	3·6–9·4	
Uterus area (UA; mm^2^)	2·8 ± 0·1	3·0 ± 0·1	2·4 ± 0·1	0·002
	1·1–5·0	1·7–4·4	1·1–4·3	
Body length (BL; mm)	5·7 ± 0·1	6·5 ± 0·1	5·8 ± 0·1	<0·001
	4·2 – 7·6	4·5 – 8·5	4·3 – 7·2	

Values shown are mean ± s.e.m. and range.

**Fig. 1.**
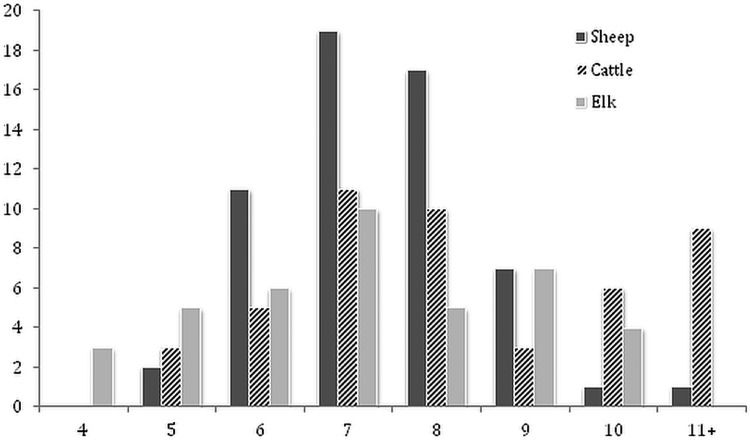
Frequency distribution of body surface area for individual *Dicrocoelium* from experimentally infected sheep (*N* = 80), cattle (*N* = 47) and elk (*N* = 39).

The frequency distributions of BA data for the total sample of flukes from each host species indicated that a high proportion of flukes from elk (0·77 ± 0·08) and from sheep (0·86 ± 0·09) were <8 mm^2^. In comparison, a higher proportion (0·38 ± 0·14) of flukes had a BA ⩾ 9 mm^2^ in cattle (Fig. 1). Overall, there was a significant difference in mean fluke BA between the three species of host (Table 2). *Post hoc* comparisons showed that flukes in cattle were on average, 14% larger than those in sheep and elk. However, results from a nested ANOVA (Table 3) showed that host species only accounted for 10·7% of the overall variation in fluke BA, while most of the variation was due to differences between host individuals (44·6%; *F*_2,152_ = 19·7, *P* < 0·001) and between individual flukes (44·7%).

**Table 3.** Nested ANOVA of the effects of host species (sheep, cattle and elk) and individual animal combinations on variation in fluke body area (mm^2^) and *ex vivo* egg count for flukes incubated for a 24 h period

	Source	d.f.	MS	*F-*value	*P-*value	Variance component	%
BA	Species	2	25·0	19·7	<0·001	0·3	10·7
	Individual hosts (Species)	10	17·3	13·7	<0·001	1·3	44·6
	Residual	152	1·3			1·3	44·7
	Total	164					
*Ex vivo* egg count	Species	2	1·3 × 10^7^	11·5	<0·001	3·6 × 10^5^	19·8
	Individual hosts (Species)	12	4·6 × 10^6^	3·9	<0·001	2·7 × 10^5^	15·2
	Residual	172	1·2 × 10^6^			1·2 × 10^6^	65·0
	Total	186					

### Comparative fluke reproduction

Mean EPG for samples collected on the date of necropsy significantly differed (*t*_20_ = 3·36, *P* < 0·01) among sheep (*x* = 38·5 ± 6·4) and cattle (*x* = 8·2 ± 3·3), with values approximately 80% higher in sheep.

**Fig. 2.**
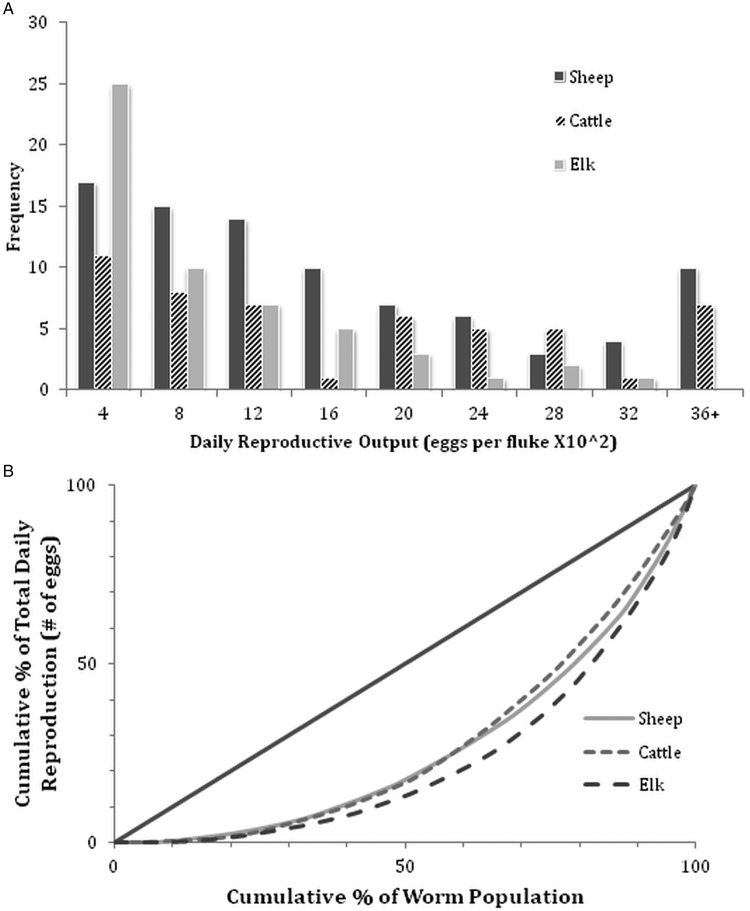
(A) Frequency distributions of daily reproductive output for individual *Dicrocoelium dendriticum* collected from experimentally infected sheep (*n* = 86), cattle (*n* = 51) and elk (*n* = 54). (B) Lorenz curves for cumulative per cent (%) of daily reproductive output of flukes plotted against the cumulative number of individual flukes. Pairing of the cumulative per cent of the fluke population responsible for a cumulative per cent of total daily reproduction in each species of hosts is achieved after ranking the flukes from least to most fecund. The solid line represents the line of equality (*G*_*R*_ = 0).

All recovered flukes were gravid. Each fluke contained hundreds to thousands of eggs *in utero*. Variation in egg output over the 24 h incubation period (Fig. 2A) spanned three orders of magnitude (range = 1–5820 eggs per day per fluke). In elk, approximately 72% (95% CI = 60–84%) of flukes shed fewer than 1000 eggs in 24 h, of which 14% (95% CI = 5–24%) shed fewer than 100 eggs. In contrast, only 44% (95% CI = 34–55%) and 45% (95% CI = 31–59%) of flukes from sheep and cattle, respectively, shed fewer than 1000 eggs over the 24 h incubation period. Approximately 14% (95% CI = 5–23%) of flukes from sheep and cattle shed more than 3000 eggs in a day, while less than 2% (95% CI = 0–5%) of flukes harvested from elk shed such high numbers of eggs.

Estimates of mean egg production/day varied significantly among host species for flukes harvested from sheep (*x* = 1530·0 ± 147·3), cattle (*x* = 1561·1 ± 173·7) and elk (*x* = 701·4 ± 98·6). Tukey's *post hoc* comparisons showed that mean *ex vivo* egg counts did not significantly differ among sheep and cattle (*P*_CS_ = 0·94), while counts for flukes harvested from elk were significantly lower (*P*_CE_ < 0·001; *P*_SE_ < 0·001). However, results from a nested ANOVA (Table 3) showed that host species and host individual only accounted for 19·8 and 15·2% of the variation in egg counts, respectively, while 65·0% of the variation could be explained by differences between individual flukes (*F*_12,175_ = 3·9, *P* < 0·001).

Since only a small proportion of the overall variation in fluke fecundity could be attributed to host species and host individuals, it is likely that inherent variation among individual flukes plays a significant role. Overall, reproductive inequalities were similar among fluke infrapopulations in cattle (G'_RC_ = 0·44, 95% CI = 0·37–0·52), sheep (G'_RS_ = 0·47, 95% CI = 0·39–0·52) and elk (G'_RE_ = 0·53, 95% CI = 0·44–0·59) with no apparent differences between host species (Fig. 2B). These results indicate that inequalities in reproduction arise from a small number of highly fecund flukes within each infrapopulation. If these data are assumed to represent random samples of flukes from the three host species, the Lorenz curves indicate that approximately 10% of flukes in any infrapopulation contribute about 30–35% of all eggs shed by a particular host. Lastly, estimates of total daily egg output using *ex vivo* egg counts (sheep: 26·4 ± 8·5 × 10^4^ eggs day^−1^; cattle: 23·6 ± 8·1 × 10^4^ eggs day^−1^; and elk: 16·4 ± 5·9 × 10^4^ eggs day^−1^) did not significantly differ among host species (*F*_2,15_ = 0·23, *P* = 0·79).

## DISCUSSION

Our data show that rates of recruitment of adult *D. dendriticum* from metacercariae in ants were approximately equivalent between at least two species of sympatric host. The rates of fluke development and time to reproductive maturity were also approximately equal between species of host. Virtually all flukes, in all hosts, reached maturity, although rates of *per capita* fecundity were highly variable. The evidence for relatively equivalent fluke performance among hosts is strongest for flukes assessed from experimentally infected sheep and cattle, where confounding factors such as dose and fluke age were controlled. The similarities in overall fluke performance were also consistent in naturally infected elk, with heterogeneity among individual flukes accounting for >50% of the total variation in reproductive performance and morphology among the fluke infrapopulations. Host-related differences were also absent in comparisons of estimated total daily egg output calculated using *ex vivo* egg counts. Taken together, these data show that *D. dendriticum* performance is approximately equivalent in all three species of hosts, two of which (cattle and elk) are sympatric within a known site of introduction in Alberta, Canada (Goater and Colwell, 2007; Beck *et al.* 2014).

Our results from the experimental infections confirm those from prior experimental work involving other domestic hosts. Manga- González and González Lanza (2005) exposed lambs to 1000–3000 metacercariae and then monitored rates of fluke recruitment, growth and fecal egg production. Our results involving lambs and beef cattle were consistent with the results of this study relative to fluke recruitment, pre-patency period and fluke size. Similarly, results indicating relatively equal performance among host species are consistent with data from field studies documenting similarities in fluke burden and fecal egg counts in sheep and goats (Jithendran and Bhat, 1996). These results indicate consistency in fluke performance between host species that are amenable to experimental exposures (e.g. sheep and cattle). Although our data from flukes collected from naturally infected elk are less conclusive, patterns of overall fluke size and *per capita* reproduction are consistent with those from the two domestic hosts.

Our analyses also indicate that fluke performance was not density dependent. The growth and survival of adult *D. dendriticum* was independent of the numbers of flukes in individual hosts. There was no detectable increase in *per capita* fecundity with fluke size in experimentally infected sheep or elk, although this relationship was moderately positive in cattle. However, fluke performance did not decrease with an increase in fluke intensity in any of the three host species. These results were unexpected, particularly when overall variation in intensity spanned four orders of magnitude. Density dependence is a frequent outcome within many host–parasite interactions (Shostak and Scott, 1993), including for the trematode *F. hepatica* in rats (Valero *et al.* 2006) and for the nematode *Trichostrongylus columbriformis* in sheep (Dobson *et al.* 1990). The absence of density dependence may be related to the small size and hence, minimal nutrient requirements of individual flukes. The minor hepatocellular damage associated with infection may also not be sufficient to activate density-dependent immunity. Density-dependent fluke performance may be evident at the higher intensities observed within some hosts (especially deer) sampled in Cypress Hills Park (up to 5000 per liver (Goater and Colwell, 2007)) and other geographic regions [up to 10 000 per sheep on the Isle of Coll, Scotland (Sargison *et al.* personal communication)]. Threshold density-dependent dynamics have been documented for *T. columbriformis* in sheep, in which decreased fluke fecundity was restricted to sheep that contained more than 3000 flukes (Dobson *et al.* 1990).

Further evidence for the absence of strong density-dependent constraints on development and survival comes from the observation that virtually all flukes, within all infrapopulations, were gravid. All flukes from the three host species that were sampled for morphological assessments contained eggs *in utero* and all randomly selected individuals that were incubated within growth media produced eggs over 24 h. This pattern of reproduction was consistent for infrapopulations in sheep and cattle, where factors such as fluke age, host age and intensity were controlled and also for infrapopulations in naturally infected elk. Although our data show that the numbers of eggs released by individual flukes was highly variable within and between individual hosts, the onset of egg release 7–12 weeks p.i., and the probability of reaching reproductive maturity, was not. These results are in striking contrast to the patterns of reproduction that are reported in other vertebrate/helminth interactions, in which reproductive inequalities within infrapopulations tend to be much higher (Keymer *et al.* 1983; review by Dobson, 1986). Shostak and Dick (1987) showed that, on average, only 33% of individual cestodes (*Triaenophorus crassus*) collected from infrapopulations in pike (*Esox lucius*) were gravid, and only a fraction of these shed eggs *ex vivo*. Overall, 10% of these gravid flukes produced 85% of the total eggs shed. Similar examples involving other host/parasite interactions are described in Dobson (1986). In contrast, the most fecund 10% of *D. dendriticum* individuals sampled from the three hosts produced only 30–35% of the total eggs shed, and all flukes contributed eggs. These results suggest that the rates of development, growth and reproduction of *D. dendriticum* in the biliary system of its definitive hosts are less constrained by factors such as inter- and intraspecific competition for host resources and/or by host responses. The implications of this contrasting pattern of fluke development and especially reproduction to patterns of population genetic structure and evolutionary potential provide interesting follow-up opportunities.

Taken together, the general transmission strategy of adult *D. dendriticum* is one of low host specificity, approximately equivalent *per capita* fluke performance in different hosts, density-independent growth and reproduction, and modest reproductive inequality between individual flukes. In effect, this means that almost every metacercariae that is recruited into the liver of a wide range of host species will develop to maturity and release large (albeit variable) numbers of viable eggs onto shared pasture. Why might natural selection favour a generalist life-history strategy for *D. dendriticum*? One possibility is that low host specificity optimizes overall rates of transmission for trematodes such as *D. dendriticum* that complete all of their life-cycle stages within terrestrial habitats. Thus, optimal rates of host exploitation of flukes that are recruited within the livers of several potential hosts may be one solution to offset the low probability of transmission during the various stages of the *D. dendriticum* life cycle (Poulin, 2007). Comparable examples involving other terrestrial or semi-terrestrial parasites (e.g. fasciolid trematodes, trichinellid nematodes, and some taeniid cestodes) tend also to be broad host generalists during their adult stages (Goater *et al.* 2014).

An alternative explanation is that exploitation by these small flukes within the bile ducts of large livers provides optimal quality and quantity of host resources to support high fluke fecundity, unencumbered by inter-specific or intra-specific competition (Poulin, 2007). This scenario may occur among sympatric grazing mammals in CHP where parasite intensities tend to be relatively low, and there are no co-occurring parasites in the livers (Goater and Colwell, 2007; Beck *et al.* 2014). A related possibility is that the liver of these ungulates may provide a relatively immuno-privileged microhabitat that does not constrain the growth and fecundity of *D. dendriticum* in potential definitive hosts. Evidence from serological assays involving experimentally infected mammals has shown that anti-*D. dendriticum* antibodies are detectable at approximately 60 days p.i. in sheep (González-Lanza *et al.* 2000; Ferreras-Estrada *et al*. 2007). However, this immunological response may not provide adequate protection, as is apparent among sheep chronically exposed to *F. hepatica* (Pérez *et al*. 2002) and cattle experimentally infected with *Fasciola gigantica* (Molina and Skerratt, 2005). Analyses of age-intensity patterns of infection in cattle sampled from CHP are also consistent with a lack of effective host immunity (Beck *et al*. 2014). These results indicate that effective host defences are minimal, absent or at least slow acting for many potential hosts of *D. dendriticum*. Given the role of host defence in selecting for host specificity across a range of host/parasite interactions (Combes, 2001; Poulin, 2007), the absence of effective anti-*D. dendriticum* immunity may best explain the patterns of host utilization and parasite performance observed in this study.

The ability of *D. dendriticum* to attain approximately equal fitness within a range of definitive hosts likely enhances opportunities for host encounter and for host switching, and likely also increases overall rates of dispersal (Combes, 2001; Hoberg and Brooks, 2008). Since this fluke utilizes a notoriously high number of species of terrestrial snail and formicid ants as first and second intermediate hosts (review by Manga-González *et al.* 2001), respectively, such opportunities likely extend through the entire life cycle. Ultimately, a generalist strategy of this kind – one that extends throughout the various life-cycle stages – will influence rates of colonization, including into novel geographical areas and naïve host populations. This is consistent with historical and contemporary patterns of host switching and the translocation of *D. dendriticum* outside its native range in Europe into areas in North and South America, Northern Europe, North Africa and the Middle East (review by Otranto and Traversa, 2002; Goater and Colwell, 2007; Rojo-Vásquez *et al*. 2012). Combined with rapid environmental change and increased mixing of wildlife and domestic stock as a result of anthropogenic changes in landscape use (review by Agosta *et al.* 2010), the reproductive strategy of *D. dendriticum* may increasingly facilitate the formation of novel host–parasite associations.
